# Competing values, conflicting views: evidence and debate in Alzheimer's disease

**DOI:** 10.1002/14651858.ED000180

**Published:** 2026-10-15

**Authors:** Toby Lasserson, Rupa Sarkar

**Affiliations:** Cochrane


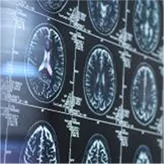


‘…since the general or prevailing opinion on any subject is rarely or never the whole truth, it is only by the collision of adverse opinions, that the remainder of the truth has any chance of being supplied.’ John Stuart Mill [Bibr ED000180-bib-0001]

As scientific editors we make decisions about the scope, impact and quality of the research that we publish. Few of us do so expecting what we publish to be the final word. One of the earliest lessons we learned about preparing systematic reviews was that they are eminently criticisable. You have to be clear about what you did, how you did it and why. Doing so lays bare your assumptions and the decisions you made in implementing your protocol methods. Transparency informs debate and rarely ends it.

The Cochrane review of **Amyloid‐beta‐targeting monoclonal antibodies for people with mild cognitive impairment or mild dementia due to Alzheimer's disease** published in April this year [Bibr ED000180-bib-0002], transparently set out its methods, analyses and conclusions. Doing so has enabled others to examine and challenge them. The review concluded that, compared with placebo, treatment with monoclonal antibodies made little meaningful difference to the decline in cognitive function, with an average of 0.85 points on the Alzheimer's Disease Assessment Scale‐Cognitive Subscale (ADAS‐Cog) after 18 months [Bibr ED000180-bib-0003]. Treatment also increased risks of brain swelling (from 1 to 30 events per 1000 people) and brain bleeds (from 2 to 7 events per 1000 people). The review has received extensive coverage in broadcast and print media. It has also been the subject of critical letters and commentaries published in the pages of other journals and on charity websites [Bibr ED000180-bib-0004 ED000180-bib-0005 ED000180-bib-0006].

Critics have as much right to challenge the findings as authors do to defend their research. Not all criticism should be dismissed as flawed or because it is biased by commercial interest. After all, disagreement is part of how human knowledge has progressed over centuries. From astronomy to stomach ulcers, consensus has emerged as evidence accumulated. However, some debates endure not in the absence of evidence, but because the evidence itself is contested and requires careful interpretation.

In this case, debate is a useful way to test different interpretations of research evidence. Readers should weigh criticism and rebuttal alongside their own perspective on the evidence. We have published one recent comment on the anti‐amyloids review as a Letter to the Editor accompanied by a response from the authors of the review [Bibr ED000180-bib-0007]. Our decision to publish this letter was driven by its novel argument about the role of biomarkers and reference to harms, alongside more familiar criticisms, such as the decision to combine data across different medicines as a class effect. The Cochrane Library has historically published comments and responses alongside reviews, but we believe that response to criticism on a review as topical as this should be citable and more visible to users.

The letter from Van Weehaeghe et al highlights three points of contention shared with other published criticism: the synthesis of data from studies testing medicines with varying degrees of success in amyloid clearance conceals true differences between them; the interpretation of the effect on cognitive function is overly negative; and the review has ignored differences in patient selection between older and newer trials. The review authors, Nonino et al, counter that the combination of data is justified by their shared mechanistic target; that the presentation of results enables transparent inspection of findings for individual antibodies; and that the effect observed on cognitive function, whether considered as a class effect or by individual antibody, is lower than the threshold for a clinically important difference.

We believe that this exchange adds value to the published review. The letters also cover ground that has featured less visibly in earlier commentaries. Nonino et al comment on the relationship between amyloid clearance and clinical outcomes, what is known of the long‐term effects, the monitoring burden, and what observational evidence can tell us about harms. Through this we learn more about the amyloid hypothesis, the contested value of biomarkers and uncertainties that still need exploration.

Publishing criticism alongside rebuttal reflects our confidence in the review and the value of scrutiny by the wider scientific community. We remain of the opinion that the review is a robust and independent summary of evidence in an area where therapeutic options are limited [Bibr ED000180-bib-0008]. As with all Cochrane reviews, it makes clear what the available evidence can tell us and where uncertainties remain. We encourage you to reflect on both letters and draw your own conclusions.

Publishing this exchange is about more than hosting different points of view. It reinforces a difficult truth. For people living with cognitive impairment and their families, treatment decisions are far from straightforward to navigate. The small average benefit in slowing cognitive decline has to be traded against repeated hospital visits for scans and the risk of treatment‐related brain swelling and bleeds [Bibr ED000180-bib-0009]. Beyond the challenges faced by patients and their families, health systems have to meet the upfront costs of treatment and establish surveillance protocols to monitor harms [Bibr ED000180-bib-0010]. When we are left with such delicate trade‐offs, competing values inevitably emerge and opinions collide.

We can measure benefit, harm and cost, but evidence alone does not make decisions. Judgement is needed. Debate helps us consider the implications of evidence, but the question that matters most is whether and how that evidence leads to better, safer care for patients. In a contested area such as this, we are unlikely to have read the final word.

## End Notes

**Declarations of interest**

TL is employed by Cochrane as Deputy Editor in Chief.

RS is employed by Cochrane as Editor in Chief.

**Acknowledgments**

The authors would like to acknowledge and thank the Cochrane Editorial Board for their support during the development of this editorial. The authors would also like to acknowledge and thank Denise Mitchell (Senior Production Editor, Cochrane) for copy editing, and Justin Mann (Managing Editor, Cochrane) for managing the editorial process.

**Provenance and peer review**

This editorial was originated by the Editor in Chief and was not externally peer reviewed.
